# Experience with the delegation of anaesthesia for disbudding and castration to trained and certified livestock owners

**DOI:** 10.1186/1746-6148-10-35

**Published:** 2014-02-04

**Authors:** Maher Alsaaod, Marcus G Doherr, Deborah Greber, Adrian Steiner

**Affiliations:** 1 Clinic for Ruminants, Vetsuisse Faculty, University of Berne, Berne 3012, Switzerland; 2 Veterinary Public Health Institute, Vetsuisse Faculty, University of Berne, Berne 3012, Switzerland

## Abstract

**Background:**

Anaesthesia is mandatory for disbudding and castrating calves and lambs of any age, in Switzerland. According to the “anaesthesia delegation model” (ADM), anaesthesia for disbudding calves <3 weeks of age and castrating calves and lambs <2 weeks of age may be administered by certified farmers. Experience with this unique model is not available. The aim was to evaluate the experience of the veterinary practitioners with the ADM. The response rate was 42%. The survey consisted of one questionnaire for each procedure. Procedure I was the delegation of anaesthesia for disbudding calves and procedures II and III were anaesthesia for castrating calves and lambs.

**Results:**

Procedure I was performed with local anaesthesia in all farms of 51.8% of the veterinary practices, while this was only 39.3% and 7.6% for procedures II and III (p < 0.001). Anaesthesia for procedure I was administered technically correctly by farmers in at least 66% of the farms of 58.3% of the practitioners, while this was 45.4% and only 23.6% for procedures II and III (p < 0.001). The ADM was assessed as a moderate to very good model to reinforce the legal obligations for procedures I, II, or III by 74.8%, 76.5% and 62.0% of the veterinary practitioners (p < 0.005).

**Conclusions:**

The delegation of anaesthesia to certified farmers may be a promising model to reinforce the obligation to provide local anaesthesia for disbudding and castrating calves, but to a lesser extent for castrating lambs.

## Background

In the United States (US), the number of castrations in male calves amounts to approximately 15 million procedures per year [1], and nearly 4 million calves are dehorned annually [2]. In New Zealand and the United Kingdom (UK), more than half a million and over one million of calves respectively, are castrated annually between the ages of two to four months [3,4]. More than 25,000 male calves under two weeks’ old are castrated in Switzerland [5] and approximately 250,000 calves under 3 weeks’ old are disbudded every year.

The advantages of performing castration and disbudding in cattle include reducing stress and aggressive behaviour, decreasing the risk of physical injury to other pen mates or stockpersons during routine management practices or veterinary examinations, and preventing unwanted breeding [4,6,7]. In lambs, preventing uncontrolled mating and reduction of abnormal sexual activity in flocks of male lambs were described as the main reasons for castration [8,9].

Castration and dehorning are painful interventions that have been under the scrutiny of public opinion and nongovernmental organisations for several years. The application of local anaesthesia alone or combined with systemic analgesic drugs for routine painful interventions is crucial to alleviate pain and to improve overall welfare [10,11]. The legal standards for administrating anaesthesia prior to castrating or dehorning livestock vary considerably among countries, depending on the technique involved and the age of the animals at which these procedures are being conducted. In the US, there are no legal standards to mitigate pain for dehorning and castration. Only 22% of all castrations of male calves that are carried out in the US by veterinarians were performed under systemic or local analgesia [1]. In contrast, numerous European countries and New Zealand have implemented animal welfare legislation that regulate pain mitigation for dehorning and castration. In the UK, the castration of male calves up to 2 months and lambs up to three months of age is permitted without anaesthesia and may be performed by lay people [3,12]. Disbudding calves using a caustic paste is permitted without anaesthesia up to the of age of 1 week, while disbudding of older calves or dehorning any cattle must be carried out under local anaesthesia administered by veterinarians [3]. In New Zealand, cattle over 6 and 9 months of age legally require analgesia before castration and dehorning, respectively [13]. In Switzerland, castrating and disbudding male calves and small ruminants of any age are only permitted after administration of local anaesthesia (article 65 of the by-law on animal protection; Art. 65, TSchV).

Although keeping and using local anesthetics may be restricted to veterinarians in some countries [14], access to and the use of analgesics by farmers under veterinary supervision have increased in the recent years [15]. In a survey including 113 midwestern and eastern US dairies, anaesthetics for dehorning cattle were used by 12.4% and analgesics by 1.8% of dairy owners. In a survey including 639 Italian dairies, 10% of the farmers used local anaesthesia before cauterization, and 5% of the farmers provided calves with postoperative analgesia [14].

In 2008, the “anaesthesia delegation model” (ADM) was introduced as a law in Switzerland: Local anaesthesia with lidocaine for disbudding calves and castrating calves and lambs less than 3 and 2 weeks of age respectively, was no longer restricted to veterinarians, but may be administered by trained and certified farmers to their own livestock only. Certification is acquired by farmers after the following 3 steps are fulfilled: (i) successful participation in a 4-hour theoretical course (this course offers a theoretical background concerning pain control for routine management procedures), (ii) practicing the procedure under the supervision of the contract veterinarian and (iii) receiving permission from the respective cantonal veterinary authority.

Delegation of anaesthesia by practitioners to farmers is practiced on a voluntary basis in some countries [14,16], but to our knowledge the ADM is unique as it is classified as a legal measure by the Swiss Animal Welfare Act. However, experience with this model and information on the actual application is not currently available. Therefore, the present survey aims to evaluate the experience of the 410 registered members of the Association of Swiss Ruminant Practitioners (SVW) with the ADM four years after its initiation. The collected information should support legislation-makers and practitioners in their decision-making process for implementing local anaesthesia for painful zoo-technical interventions in calves and lambs.

## Methods

### Survey design

In the fall of 2012, a survey was conducted among all SVW members to validate the ADM. In order to include only ruminant practitioners, each practitioner had to declare the percentage of working time spent in the livestock sector. Ruminant practitioners were defined as veterinarians that dedicated at least 50% of their working time to livestock of which at least 80% were ruminants. Practitioners not fulfilling these conditions were excluded from the survey. Only one practitioner per practice was allowed to fill in the questionnaires. Information concerning the survey, including a link to the electronic versions of the questionnaires on the web-based online survey tool “Survey monkey” was posted in an electronic newsletter and sent twice at an interval of 2 weeks to all members of the SVW by E-mail. Additionally, a paper copy of the questionnaires was sent to each member of SVW by letter post. It was left up to the discretion of the individual practitioner to fill in and submit either the electronic (n = 79; 46.5%) or the paper (91%; 53.5%) version. The questionnaires were made available in German and French.

### Design of questionnaires

The survey consisted of one questionnaire for each of the three procedures, composed of 8 similar questions. Procedure I was disbudding calves less than 3 weeks’ old; procedure II was castrating calves less than 2 weeks’ old, and procedure III was castrating lambs less than 2 weeks’ old. One question (Number 6) was excluded from the evaluations, since it did not add any valuable information to the survey. For each question, a set of five categorized answers were provided, and respondents were expected to choose the single best fitting answer to each question. The question texts and the proportion of answers by response category are given in Table 1.

**Table 1:** Responses to the seven questions asked in a survey on “experiences with the delegation of anaesthesia for disbudding and castration to trained and certified livestock owners” in Switzerland (2012)

**Question 1: In my client farms, in which procedures I, II or III are conducted, anaesthesia is still administered by vets of my practice. This applies to .% of my client farms.**						**Statistical comparison (p-values of cross tabulation and Pearson’s chi square test)**			
**Answers**	**0%**	**1-33%**	**34-66%**	**67-99%**	**100%**	**vs proc I**	**vs proc II**	**vs proc III**	**overall**
**Procedure I**	2.5%	28.8%	25.2%	35.0%	8.5%			< 0.0001	
**Procedure II**	2.4%	27.1%	18.7%	36.1%	15.7%	0.279			
**Procedure III**	26.5%	46.3%	14.3%	8.2%	4.7%		< 0.0001		
**Procedures I-III**									< 0.0001
**Question 2: As compared to the situation prior to the introduction of the ADM, my practice now performs …. anaesthesia for procedures I, II or III in my client farms.**									
**Answers**	**Clearly less**	**Less**	**Equal**	**More**	**Clearly more**	**vs proc I**	**vs proc II**	**vs proc III**	**overall**
**Procedure I**	11.3%	25.0%	39.4%	10.6%	13.7%			0.007	
**Procedure II**	9.4%	26.9%	44.4%	8.8%	10.5%	0.774			
**Procedure III**	18.0%	18.0%	48.2%	12.2%	3.6%		0.012		
**Procedures I-III**									0.022
**Question 3: In my client farms, procedures I, II or III are still performed without anaesthesia. This applies to …% of my client farms.**									
**Answers**	**0%**	**1-33%**	**34-66%**	**67-99%**	**100%**	**vs proc I**	**vs proc II**	**vs proc III**	**overall**
**Procedure I**	51.8%	41.4%	5.6%	1.2%	0.0%			0.0001	
**Procedure II**	39.3%	50.9%	6.7%	3.0%	0.0%	0.122			
**Procedure III**	7.6%	38.6%	29.7%	23.4%	0.7%		0.0001		
**Procedures I-III**									0.0001
**Question 4: In my client farms, external certified lay person administer anaesthesia for procedures I, II or III. This applies to …% of my client farms.**									
**Answers**	**0%**	**1-33%**	**34-66%**	**67-99%**	**100%**	**vs proc I**	**vs proc II**	**vs proc III**	**overall**
**Procedure I**	49.4%	43.1%	4.4%	3.1%	0.00%			0.143	
**Procedure II**	66.1%	30.9%	1.8%	1.2%	0.00%	0.018			
**Procedure III**	45.7%	40.7%	11.4%	2.2%	0.00%		< 0.0001		
**Procedures I-III**									< 0.0001
**Question 5: My experience with the ADM showed that my certified clients administer the anaesthesia for procedures I, II or III technically correct. This applies to …% of my client farms.**									
**Answers**	**0%**	**1-33%**	**34-66%**	**67-99%**	**100%**	**vs proc I**	**vs proc II**	**vs proc III**	**overall**
**Procedure I**	3.8%	16.7%	21.2%	52.5%	5.8%			0.001	
**Procedure II**	12.9%	22.7%	19.0%	37.4%	8.0%	< 0.0001			
**Procedure III**	12.5%	36.1%	27.8%	22.2%	1.4%		0.001		
**Procedures I-III**									< 0.0001
**Question 7: Some certified farmers realised that administration of anaesthesia for procedures I, II, or III is a demanding task and, therefore, continued to assign their contract veterinary practice to administer anaesthesia. This applies to …% of my client farms.**									
**Answers**	**0%**	**1-33%**	**34-66%**	**67-99%**	**100%**	**vs proc I**	**vs proc II**	**vs proc III**	**overall**
**Procedure I**	17.8%	51.4%	19.8%	9.6%	1.4%			< 0.0001	
**Procedure II**	15.6%	50.4%	19.8%	12.8%	1.4%	0.587			
**Procedure III**	50.4%	31.3%	14.8%	3.5%	0.00%		< 0.0001		
**Procedures I-III**									< 0.0001
**Question 8: I assess the ADM as … model to reinforce the legal obligation to administer local anaesthesia for procedures I, II, or III.**									
**Answers**	**Very poor**	**Poor**	**Moderate**	**Good**	**Very good**	**vs proc I**	**vs proc II**	**vs proc III**	**overall**
**Procedure I**	6.8%	18.4%	28.8%	35.0%	11.0%			0.004	
**Procedure II**	6.2%	17.3%	40.7%	29.0%	6.8%	0.202			
**Procedure III**	15.2%	22.8%	35.1%	22.8%	4.1%		0.043		
**Procedures I-III**									0.005

vs = versus; procedure I = anaesthesia for disbudding of calves less than 3 weeks of age; procedure II = anaesthesia for castration of calves less than 2 weeks of age; procedure III = anaesthesia for castration of lambs less than 2 weeks of age; vs = versus; ADM = anaesthesia delegation model; significance level for overall test: p < 0.05; significance level for pairwise comparisons was adjusted according to Bonferroni: p_adj_ < 0.0167.

### Statistical methods

Data were exported into MS Excel (http://www.microsoft.com) and the statistical package NCSS (http://www.ncss.com) for further processing and validation. Cross tabulation and Pearson’s chi-square tests were performed to compare the distribution of answers (proportions of checked answer classes) of corresponding questions among procedures I to III. In questions 3, 4, and 7, the number of answers in classes 4 and 5 were small so that they were brought together in class 4/5 for comparisons among procedures within questions. The alpha level of significance for the overall test was set at 0.05 and adjusted for three group comparisons according to Bonferroni (alpha_adj_ = 0.0167).

## Results

A total of 170 SVW members returned the questionnaire, representing an overall response rate of 42%. Frequency distributions of the given answers for all questions and p-values for comparisons among procedures within questions are provided in Table 1. The proportion of veterinary practices in which anaesthesia for procedures I, II, or III was still conducted by practitioners in more than 66% of their contract farms was 43.5% and 51.8% for procedures I and II, but only 12.9% for procedure III (p < 0.0001 for procedure III as compared to procedures I and II). As compared to the situation before the introduction of the ADM, the number of farms in which anaesthesia was performed by practitioners diminished. While in 75.7% and 80.7% of farms, clearly less, less or equal anaesthesia were administered by practitioners for procedures I and II, respectively, this was even more notable (84.2%) for procedure III (p < 0.015 for procedure III as compared to procedures I and II). It was indicated by 51.8% of the practitioners that in 100% of their client farms, disbudding of calves was nowadays performed with local anaesthesia, while this was 39.3% for procedure II and only 7.6% for procedure III (p < 0.0001 for procedure III as compared to procedures I and II). Based on the assessment of the practitioners, external lay persons illegally administered anaesthesia for procedure I in at least some client farms in 50.6% of the responding veterinary practices, while this was 33.9% and 54.3% for procedures II and III, respectively (p < 0.0001 for procedure II as compared to procedure III). Anaesthesia for procedure I was administered technically correctly (according to good veterinary practice) by certified farmers in at least two-thirds of the client farms of 58.3% of the responding practitioners, while this was 45.4% and only 23.6% for procedures II and III, respectively (p < 0.001 between all procedures). As administration of anaesthesia was judged to being too demanding, two-thirds or more of the certified farmers assigned their contract practice to administer anaesthesia for procedure I in 11.0% of the veterinary practices. This was 14.2% for procedure II, but only 3.5% for procedure III (p < 0.0001 for procedure III as compared to procedures I and II). The ADM was generally assessed as a moderate to very good model to reinforce the legal obligation to administer local anaesthesia for procedures I, II, or III by 74.8%, 76.5% and 62.0% of the practitioners, respectively (p < 0.01 for procedure I as compared to procedure III).

## Discussion

In the survey reported here, the experience of the members of the SVW with the ADM was summarized regarding its potential to reinforce in practice the legal obligation to provide local anaesthesia for disbudding calves less than 3 weeks of age and castrating calves and lambs less than 2 weeks of age. It was shown, that the ADM was feasible for both procedures in calves, but to a considerably lesser extent for castrating lambs.

Whilst interpreting the results of this survey, it is important to be aware that the opinion and experience of veterinary practitioners but not of farmers were taken into consideration. The farmers’ attitudes were mainly assessed on the basis of the practitioners’ knowledge of the sizes of their client farms and the number of procedures performed in the respective farms, relating to the amount of anaesthetic drugs provided to these farmers. The 42% response rate of the current survey was higher as compared to that of surveys on attitudes of dairy veterinarians in New Zealand, Denmark and the UK regarding painful procedures and conditions in cattle with 37%, 28% and 27%, respectively [13,17,18] and compared to a survey on attitudes of bovine practitioners to pain and painful interventions in the feet of dairy cattle with a response rate of 27% [19]. The number of practices that were excluded, because none of practice associates met the inclusion/exclusion criteria is not known.

The rate of anaesthesia performed by practitioners considerably diminished as a consequence of the introduction of the ADM. As there is currently a shortage of members of SVW in Switzerland (http://www.srf.ch/player/radio/regionaljournal-bern-freiburg-wallis/audio/mangel-an-grosstieraerzten-besonders-in-berggebieten?id=05a86425-5a19-463b-9f8e-3e42a7f73601), similar to the situation in Germany [20] and the US (http://usatoday30.usatoday.com/news/nation/2008-02-28-vetshortage_N.htm), a reduction in the manual workload of members of SVW is not of any major economic concern for the profession. Although this represents an illegal behaviour, it is not very surprising that external certified farmers performed anaesthesia for procedures I to III in some farms, because (i) veterinarians might not always be available for this task at the time chosen by the farmer and (ii) external farmers might do the job for less money. It may be expected that the quality of the anaesthesia performed by external certified farmers would be high, as the number of procedures performed by such persons is expected to be considerably higher than the farmers which practice anaesthesia in their own livestock only. It is recommended by the Dairy Farmers of Canada and the National Farm Animal Care Council [2] that only trained persons carry out disbudding/dehorning procedures. On farms in Northern Italy, only a small proportion of the farmers had been trained for disbudding calves by specialized personnel (26.0% by a veterinarian and 4.2% by a milk quality inspector, respectively). The remaining 70% reported that they had learnt the technique on their own (26.8%) or from another farmer (43.0%) [14].

As demonstrated with this survey, a considerable proportion of anaesthesia for procedures I to III is not performed technically correctly by certified farmers. This is particularly true concerning the anaesthesia for castrating lambs. The main reason for this may be that many Swiss sheep owners are not professional farmers but rather hobby animal keepers with minor or without any basic agricultural education and training. The number of anaesthesia the majority of hobby sheep owners perform per year may not be sufficient to maintain an acceptable technical standard. Those farmers should, therefore, be advised to either waive castrating lambs or to assign their contract veterinarian to perform anaesthesia for castration. The latter would be particularly effective, as the current study showed that the number of certified sheep farmers who realised that procedure III is a demanding task, is significantly lower as compared to the certified farmers, performing procedures I and II. We speculate that the complexity of the procedure was mainly underestimated by those farmers that refused to perform anaesthesia at all, thereby not complying with the law.

Unfortunately, many painful castrations in calves and lambs and many disbuddings of calves were still performed without anaesthesia, despite the fact that this is illegal in Switzerland. Anaesthesia for procedure I was administered in all client farms of 52% of the responding practitioners. This is not much better than the current situation in Québec dairies: [21] reported the use of anesthesia for disbudding among 44.7% of the Québec dairy producers. It is, however, considerably more frequent than the reported use of local anaesthesia by dairy farmers disbudding calves in Northern Italy (10%; [14] and in the US (12.4%; [16]. The proportion of veterinary practices in which procedure II was performed under local anaesthesia by all contract farms was 39%. In a US survey among bovine practitioners, it was reported that 22% of the responding practitioners provided local anaesthesia before calf castration [1]. The proportion of procedures that is still performed without anaesthesia was significantly higher for procedure III as compared to procedures I and II. The reasons for this may be (i) the lacking professional knowledge and attitude of many hobby sheep owners and (ii) the low economic value of lambs, forcing professional producers to keep the production costs as low as possible. It was reported that lamb castration was probably not conducted with local anaesthesia, unless it was reinforced as a condition of obligatory farm insurance or by legislation [22]. The latter is not fully supported by the results of the present study.

In order to considerably increase the proportion of the procedures I-III performed under local anaesthesia, an information campaign might be undertaken to repeatedly inform stock owners that performing these procedures without anaesthesia is illegal in Switzerland and undermines animal welfare. Furthermore, they should be informed that calves treated with NSAID after disbudding were found to consume more starter feed than controls [23,24] and that negative handling could lead to fear of humans in young replacement stock [25]. Fear was shown to have potentially negative effects on milk yield in commercial dairy herds [26]. The technical quality of anaesthesia might be increased, if only farmers that perform a sufficient number of procedures were certified. Finally, we suggest that the law should be reinforced in Switzerland by more rigorous on farm veterinary controls.

The information derived from this survey may support livestock practitioners in their decision-making process to teach and delegate anaesthesia to interested stock owners in order to increase animal welfare on a voluntary basis in countries in which anaesthesia for procedures I-III is not mandatory. Legislation-makers may acquire important information on the success rate of an alternative model to reinforce the obligation to provide local anaesthesia during painful zoo-technical interventions in calves and lambs, emphasizing the essential role of education, training and communication between livestock owner and veterinary practitioner.

## Conclusions

The results of this survey show that the delegation of anaesthesia to trained and certified farmers may be a promising model for putting into practice the obligation to provide local anaesthesia for castrating and disbudding calves, but the ADM appeared to be less feasible for castrating lambs. Certification should only be granted to farmers that perform the respective procedures frequently enough to maintain the correct technique.

## Competing interests

The authors declare that they have no competing interests.

## Authors’ contributions

AS participated in the design, coordination and draft the manuscript. MA carried out the survey study and helped in drafting the manuscript. MGD carried out the statistical analysis. DG participated in the data collection. All authors read and approved the final manuscript.
